# Reflexive and Intentional Saccadic Eye Movements in Migraineurs

**DOI:** 10.3389/fneur.2021.669922

**Published:** 2021-04-07

**Authors:** Filipp M. Filippopulos, Christine Goeschy, Florian Schoeberl, Ozan E. Eren, Andreas Straube, Thomas Eggert

**Affiliations:** Department of Neurology, German Center for Vertigo and Balance Disorders-DSGZ, University Hospital of the Ludwig-Maximilians-University Munich, Munich, Germany

**Keywords:** migraine, oculomotor deficits, reflexive saccades, intentional saccades, spatial remapping

## Abstract

**Background:** Migraine has been postulated to lead to structural and functional changes of different cortical and subcortical areas, including the frontal lobe, the brainstem, and cerebellum. The (sub-)clinical impact of these changes is a matter of debate. The spectrum of possible clinical differences include domains such as cognition but also coordination. The present study investigated the oculomotor performance of patients with migraine with and without aura compared to control subjects without migraine in reflexive saccades, but also in intentional saccades, which involve cerebellar as well as cortical networks.

**Methods:** In 18 patients with migraine with aura and 21 patients with migraine without aura saccadic eye movements were recorded in two reflexive (gap, overlap) and two intentional (anti, memory) paradigms and compared to 25 controls without migraine.

**Results:** The main finding of the study was an increase of saccade latency in patients with and without aura compared to the control group solely in the anti-task. No deficits were found in the execution of reflexive saccades.

**Conclusions:** Our results suggest a specific deficit in the generation of correct anti-saccades, such as vector inversion. Such processes are considered to need cortical networks to be executed correctly. The parietal cortex has been suggested to be involved in vector inversion processes but is not commonly described to be altered in migraine patients. It could be discussed that the cerebellum, which is recently thought to be involved in the pathophysiology of migraine, might be involved in distinct processes such as spatial re-mapping through known interconnections with parietal and frontal cortical areas.

## Introduction

Migraine has been postulated to lead to functional and to lesser extend structural changes in different brain areas, such as brainstem and cerebellum (1), but also the frontal and parietal lobe (2). This hypothesis has mainly resulted from neuroimaging (3–5) and, to lesser extent, to clinical findings (6, 7). One common finding in these imaging studies is, that migraine patients seem to have structural abnormalities in the cerebellum and brainstem, although the nature of these structural abnormalities varies. Also an increase in cerebellar activity has been shown during a migraine attack; the functional importance of this activation though is widely unknown (5). Structural differences have been shown for gray matter volumes in the cerebellum with an volume increase in some studies (5), and an decrease in others (3, 4). Another interesting observation is, that migraine patients seem to have an increased prevalence of white matter lesions in the cerebellum compared to the normal population (8, 9). Further, such white matter lesions are also found in the frontal and parietal lobe of migraineurs (2), with different studies also suggesting subtle functional deficits of frontal lobe functions (10). For example, it has been suggested that patients with migraine have deficits in attentional tasks (11) and perceptual organization (global-local) tasks (12).

Overall, previous studies suggest functional abnormalities of the frontal—parietal—brainstem—cerebellar circuit possibly leading to different clinical or subclinical deficits in migraineurs.

One well-established method of evaluating the function of fronto-cerebellar pathways is through oculomotor studies on horizontal eye movements. Furthermore, intentional saccades, such as the anti-saccade, have been shown to be impaired in patients with frontal deficits (13). So far, only a few studies have investigated possible oculomotor deficits (14–17), as well as subclinical neuro-ophthalmologic pathologies (18) in patients with migraine. The spectrum of these findings seems to be wide and inconclusive. Harno et al. (18), for example, described hyper- and hypo-metric horizontal saccades in patients with migraine, while other studies showed no deficits in saccade metrics (16, 17). Latter studies though found a significantly reduced velocity gain in the smooth pursuit paradigm. Only one study investigated, besides reflexive saccadic eye movements, intentional saccades by the means of the anti-gap paradigm and found an increased number of saccade errors, suggesting a deficit in the inhibition of reflexive saccades in migraineurs (15). This in turn could support the prior neuroimaging and clinical findings of a structural and functional deficit in the activation of the prefrontal cortex leading to a decreased inhibitory function in patients suffering from migraine.

The present study investigates the oculomotor function in migraineurs with and without aura in reflexive and different intentional horizontal saccadic eye movements. Through this, the study aimed to describe characteristic changes in oculomotor function and discuss (sub-) clinical implications of a possibly disturbed saccadic network in migraineurs.

## Methods

### Subjects

In total 66 subjects participated in the study after signing informed consent. All subjects underwent a basic neurological examination. Subjects with a positive history of neurological disorders or a pathological examination were excluded from the study. After exclusion a total of 64 subjects were included.

The characteristics of the patient and control group are summarized in Table 1. The migraine group consisted of a total of 39 patients, 21 without (MO) and 18 with aura (MA). The migraine diagnosis was made according to the International classification for headache disorders (ICHD-3) (19) by a trained neurologist. All criteria had to be fulfilled for the diagnosis. Overall, the gender distribution in the migraine group was typical for a migraine population (female 2 to male 1, for details see Table 1). In the MA group only patients with visual aura were included, patients with e.g., vestibular aura or hemiplegic migraine were excluded. Migraine patients were recruited from the Upper Bavarian Headache Center, an outpatient clinic of the University Hospital of Munich.

**Table 1:** Summary of patient characteristics.

**Group**	**Age**	**Gender (f:m)**	**Disease duration (years)**	**Migraine frequency (days/month)**	**Mean migraine duration (hours)**	**Last migraine attack (days prior to exam)**
MA	37 ± 14	10:8	15 ± 12	3 ± 2	20 ± 23	31 ± 33
MO	36 ± 12	16:5	13 ± 11	5 ± 4	22 ± 22	15 ± 20
CTR	30 ± 8	14:11	-	-	-	-

*Mean ± standard deviation is displayed when applicable. Decimal MO, migraine without aura; MA, migraine with aura; CO, controls*.

The control group (*N* = 25, 15 female and 12 male) had no history of relevant medical, neurological, or psychiatric diagnoses. In particular, the control group did not suffer from any headache disorder nor had a history of recurrent headaches. All subjects of the control group did not take any regular medication. Control subjects were recruited through advertisement.

Age was not exactly matched between the age of 20-40 years, because no age-related effects on saccade performance or saccade latency are expected in this age span (20, 21).

On the recording day, all subjects (controls and patients) were headache free and had not taken any medication (e.g., non-steroidal anti-inflammatory drugs) for at least 24 h prior to the study participation. Furthermore, all patients of the migraine group were under no prophylactic treatment for at least 3 months prior to the recording day. All subjects had no prior experience regarding eye movement experiments.

The authors confirm that all methods were performed according to National Institutes of Health guidelines and in adherence with the Declaration of Helsinki. The study protocol was approved by the Data Safety Officer and the Ethics Committee of the Medical Faculty of the Ludwig-Maximilians-University Munich. Informed consent was obtained from all participants included in the study.

### Recording

Subjects were seated in front of a screen at a distance of 1.40 m. The head was stabilized to minimize movements. Targets were projected onto the screen by a red laser, controlled by mirror galvanometers (General Scanning Inc., MA, USA) in complete darkness. The eye position was recorded by a binocular, head-mounted eye tracker (22). The pupil center was computed by an online pupil detection algorithm sampled at 200 Hz. The resolution was below 0.1° and the total accuracy below 0.5°. Eye movements were recorded on a central recording system (23).

### Paradigms

**Figure 1:**
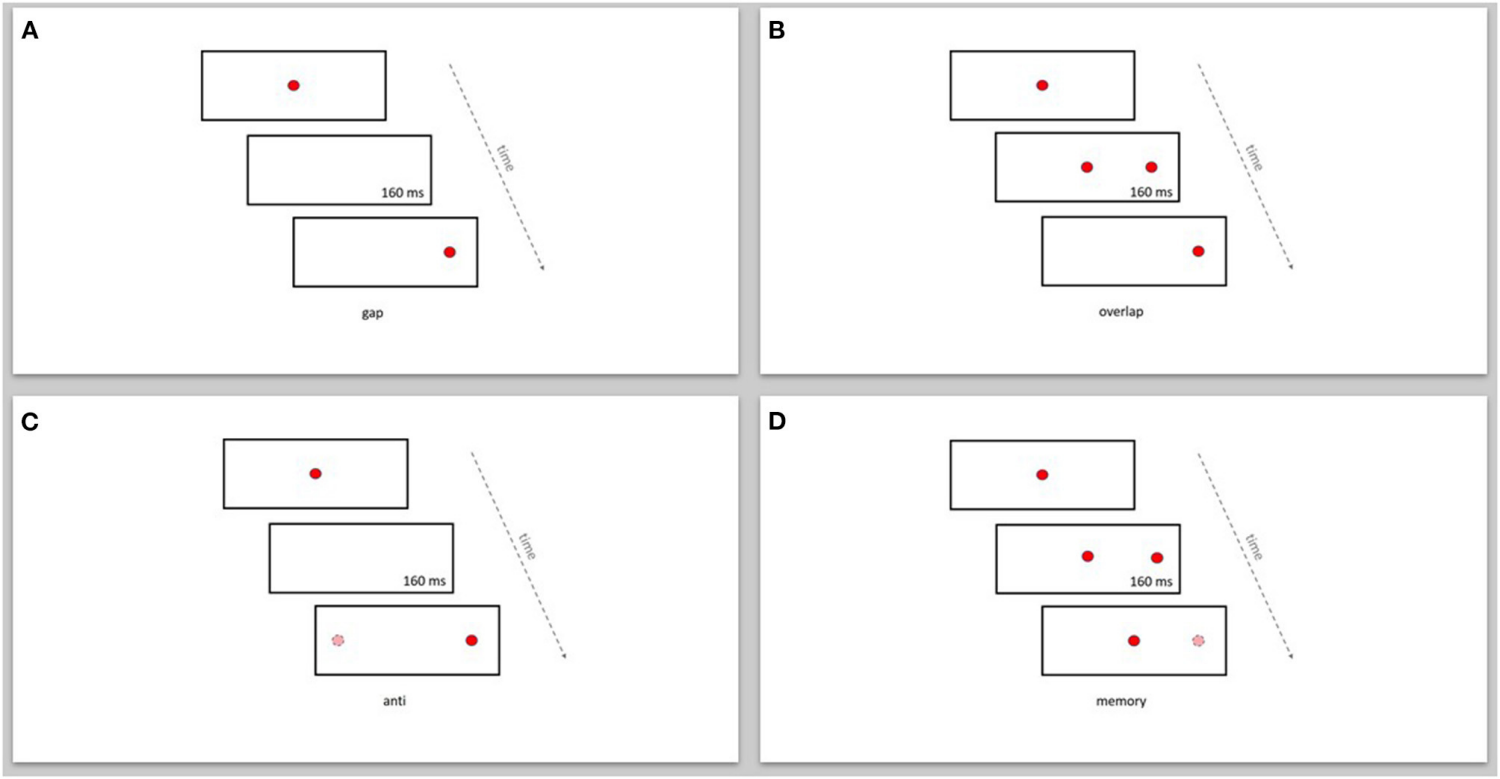
Display of the reflexive and intentional saccade paradigms. **(A)** gap, **(B)** overlap, **(C)** anti, **(D)** memory. Dark (red) circle: displayed target position. Bright (pink) circle: imagined target position.

Each patient performed two reflexive saccade paradigms (gap and overlap) and two intentional saccade paradigms (anti and memory) (Figure 1). Each patient performed about 33 trials per paradigm. The target sequence was projected in a paradigm specific pseudorandom order. All subjects were instructed to execute the saccades to the correct targets as precise as possible.

In the gap-paradigm the saccade target appeared 160 ms after the disappearance of the fixation target (see Figure 1A). In the overlap-paradigm the fixation target and saccade target overlapped for 160 ms (see Figure 1B). In both these paradigms the go-signal was the appearance of the saccade target. In the anti-paradigm the visual target appeared 160 ms after the disappearance of the fixation target. Subjects were then instructed to execute a saccade toward an imagined target in the opposite direction than the visual target, at the same distance from the fixation target (see Figure 1C). In the memory-paradigm the saccade target appeared for 160 ms while the fixation target remained on. Subjects were instructed to keep fixation on the fixation target until the go-signal (see Figure 1D). In the anti- and memory-paradigm a short interruption of the fixation target was used as the go-signal to execute the saccade.

### Data Analysis

#### Dependent Variables

The mean gain, the latency, and the percentage of errors were computed for all primary saccades. Primary saccades were defined as saccades that started closer than 3° to the initial target position, with a gain larger than 50%, and an onset time later than 80 ms after the go-signal (latency > 80 ms). The percentage of “saccade errors” was defined as the fraction of primary saccades with the wrong direction with respect to all primary saccades. In addition, for the memory-paradigm, “suppression errors” were defined as saccades toward the target that started later than 80 ms after the target flash and earlier than 80 ms after the go-signal. The percentage of suppression errors was expressed as the fraction of suppression errors with respect to all saccades (suppression errors + primary saccades).

#### Statistical Analysis

The mean gains and latencies of the executed saccades were normally distributed (Lilliefors test *p* < 0.1) and were submitted to an ANOVA with one three-level-factor Group (control, patient without aura, patient with aura). The probability of erroneous saccades was not normally distributed and was submitted to the Kruskal-Wallis ANOVA to test for overall group differences. In case that the ANOVA (parametric or non-parametric) showed significant group differences, paired differences were further evaluated by Scheffé *post-hoc*-tests (gain, latency) or the non-parametric Mann–Whitney *U*-test (probability of erroneous saccades).

Subject were classified as outliers if the latency differed more than three times the median-quartile difference from the median of the population. Alpha levels smaller than 0.05 were considered significant.

## Results

The mean gain and latency of all primary saccades, as well as the probability of the different saccade errors for each paradigm is summarized in Table 2.

**Table 2:** Summary of saccade characteristics for the reflexive and intentional saccade paradigms.

	**Paradigm**	**Group**	**Gain (deg)**	**Latency (ms)**	**Saccade errors (%)**	**Suppression errors (%)**
Reflexive	Gap	MO	0.95	153.14	0.00	n/a
		MA	0.93	144.53	0.00	n/a
		CO	0.96	151.65	0.00	n/a
	Overlap	MO	0.98	216.58	0.00	n/a
		MA	0.97	220.12	0.00	n/a
		CO	0.97	213.24	0.00	n/a
Intentional	Anti	MO	0.91	304.67	7.0	n/a
		MA	0.85	310.09	9.0	n/a
		CO	0.94	247.64	11.0	n/a
	Memory	MO	0.93	295.76	0.00	11.0
		MA	0.86	316.56	0.00	8.0
		CO	0.89	302.50	0.00	7.0

*MO, migraine without aura; MA, migraine with aura; CO, controls*.

### Gain

Overall, there were no significant differences between the mean gain in control patients compared to migraine patients. In the memory-paradigm the gain tended to differ between the three groups [ANOVA: *F*_(2, 50)_ = 2.64; *p* = 0.08]. This effect was related to a larger gain (0.93 ± 0.08) in migraine patients without aura compared to migraine patients with aura (0.86 ± 0.06; Scheffé *post-hoc*-test: *p* = 0.09). The gain of controls (0.89 ± 0.09) did not differ from that of the patients.

### Latency

**Figure 2:**
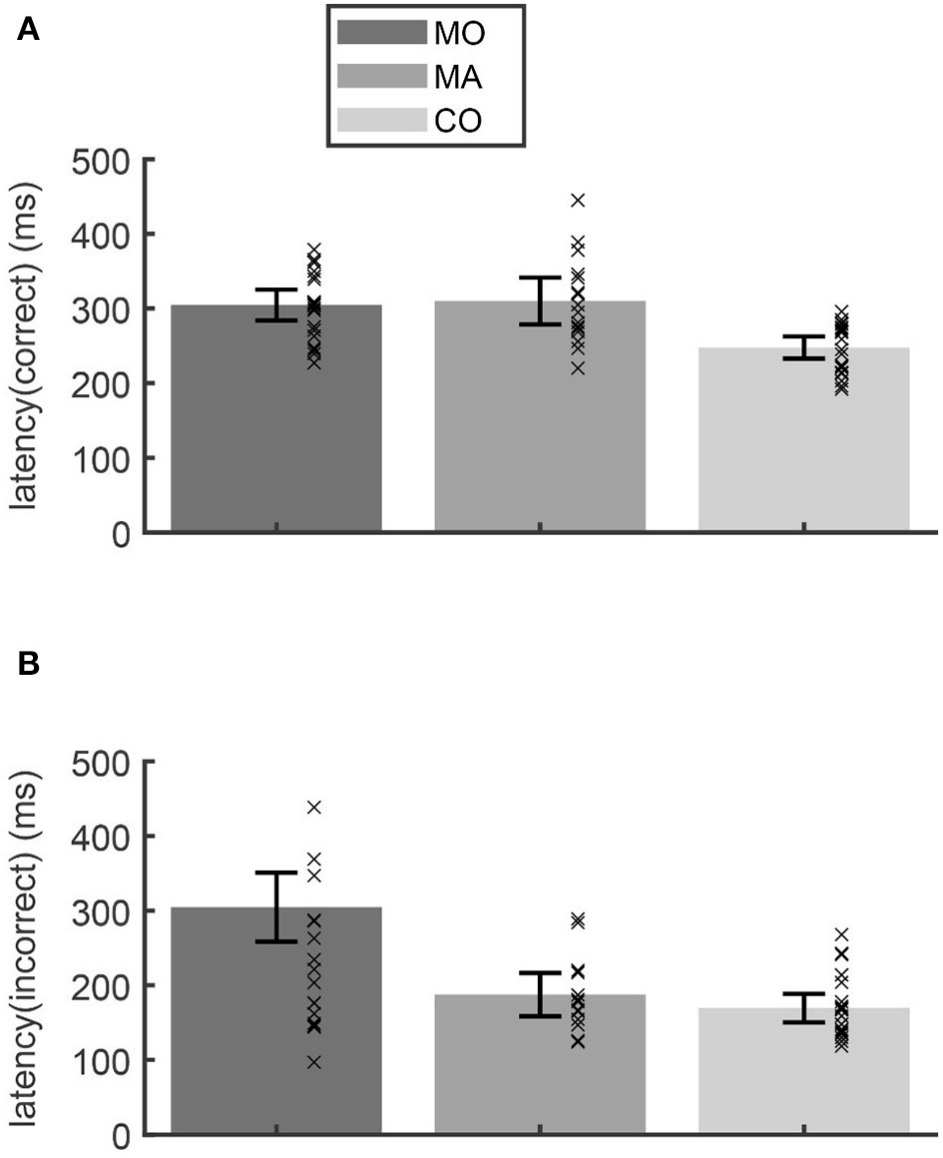
In the anti-paradigm, the latency of the correct saccades **(A)** showed a longer latency for migraine patients without (MO) and with aura (MA) compared to the control subjects (CO). The latencies of the incorrect anti-saccades **(B)** of patients without aura were larger than those of the other groups. Bars show the mean latency for each group and whiskers the 95% confidence interval of the mean. Crosses indicate the mean latency for each subject.

In the anti-paradigm saccade latencies for primary saccades toward the correctly imagined anti-target differed significantly between the groups [ANOVA: *F*_(2, 55)_ = 11.36; *p* < 0.001]. The latency was longer in patients with (310 ms) and without (305 ms) aura compared to the control group (248 ms; Scheffé *post-hoc*-test: *p* < 0.001; Figure 2A). For the latency of primary saccades toward the (wrong) visual target (Figure 2B) the group differences showed a similar tendency [*F*_(2, 50)_ = 3.16; *p* = 0.051] due to longer latencies in patients without aura (222 ms vs. controls 169 ms; Scheffé *post-hoc*-test: *p* = 0.053). In the three other paradigms, testing reflexive or memory guided saccades, the mean saccade latencies did not differ between groups (ANOVA: *p* > 0.25).

#### Correlation Between the Saccade Latency in the Anti-paradigm and Clinical Disease Parameters

To further evaluate if the longer latencies in the anti-paradigm in migraine patients correlate with different clinical disease parameters the Pearson Correlation coefficient was computed. The clinical parameters were: (a) the disease duration of the migraine, (b) the attack frequency of the migraine per month, and (c) the time between the last migraine attack and the recording day. Descriptives of these parameters are displayed in Table 1.

Results showed no correlation between the disease duration, the attack frequency, or the time of the last attack and the saccade latency of primary saccades in the correct or wrong direction (disease duration: minimal correlation coefficient *r* = −0.306, maximal correlation coefficient *r* = 0.303 over all paradigms; attack frequency: minimal correlation coefficient *r* = 0.012, maximal correlation coefficient *r* = 0.119; time of last attack: minimal correlation coefficient *r* = −0.235, maximal correlation coefficient *r* = 0.124).

### Erroneous Saccades

In gap-, overlap-, and memory paradigms, erroneous saccades (i.e., non-anticipatory, incorrect saccades) did not occur [Table 2, column saccade errors (%)] The probability of the saccade errors in the anti-paradigm and suppression errors in the memory-paradigm was about 9% and did not significantly differ between patients and controls.

## Discussion

The primary goal of the study was to investigate a possible (sub-)clinical impairment in patients suffering from migraine by studying the oculomotor performance in different horizontal saccadic eye-movement tasks. There were no differences in the metrics and the latencies of reflexive saccadic eye movements. In the intentional tasks, migraineurs (with and without aura) showed longer saccade latencies only in the anti-paradigm, while in the memory task the latencies were normal. The increased saccade latency in migraineurs in the anti-paradigm did not correlate with the disease duration, migraine attack frequency per month, or time between the last migraine attack and the recording day. With respect to controls, the patients did not show an increased probability of erroneous, non-anticipatory saccades in the anti-task, nor an increased probability of suppression errors in the memory-task.

Only few studies have investigated saccade characteristics in migraine patients. The present finding of normal saccade parameters (gain, latency, errors) in reflexive saccade tasks in patients with migraine compared to controls, independent of the presence of a migraine aura, is consistent with most published studies on saccadic eye movements in migraineurs (16, 17). Only one study found abnormal saccade metrics within the scope of a broad neurotologic testing (18). In this study ~30% of the migraine patients showed hyper- or hypo-metric saccades in reflexive saccade tasks. However, there was no consistent finding of a distinct deficit of the saccadic gain in any of the two migraine groups (with and without aura) nor aura type (visual, basilar, hemiplegic aura).

The observed tendency of increased gain of memory saccades in migraineurs without aura compared to patients with aura has not been described previously. To our knowledge there is no previous study investigating memory saccades in migraineurs, furthermore it seems that most reported (sub-) clinical oculomotor deficits are more pronounced in migraineurs with aura than in migraineurs without aura (17, 18). Therefore, the interpretation of this marginal effect is difficult, especially because we did not find any other group differences between patients and controls concerning the saccade gain. Main cortical areas involved in the generation of memory saccades are believed to be the posterior parietal and prefrontal cortex (24–26). Especially lesions in the parietal cortex have been shown to lead to dysmetric saccades (both hyper- and hypo-metric saccades), suggesting a role in visuospatial integration (25). However, this does not explain the observed tendency of a specific gain deficit in patients without aura. Overall, it seems that gain and latency of reflexive or memory saccades are not affected in patients with migraine.

To our knowledge, only one previous study investigated, besides reflexive saccadic eye movements, intentional saccades by the means of the anti-gap paradigm (15). Anti-saccades are intentional saccades that require different cognitive functions to be executed correctly. For the correct execution three main processes can be distinguished: (1) the inhibition of an unwanted reflexive saccade toward the target, (2) the generation of an internal target to the opposite site of the visual cue (vector inversion), and (3) the disinhibition of the fixation and the execution of the intentional anti-saccade.

The major finding of the present study was a significant increased latency of correct anti-saccades in migraine patients with and without aura compared to controls. Interestingly the saccade latency in the memory-paradigm was in the normal range and did not differ between migraineurs and controls. Thus, the noted deficit seems to be specific for the generation of an anti-target and does not point toward a general deficit in the execution of intentional saccades. The process, that is specifically needed to generate a correct anti-saccade is the vector inversion, meaning, the process to internally convert a saccade toward a visual cue to a saccade toward an imagined/internal target on the opposite side. A possible effect of acute or prophylactic migraine treatment on the study results was excluded by including only subject that had not taken any acute medication (e.g., NSAIDs, triptans) for at least 24 h, and no prophylactic treatment (e.g., amitriptyline, topiramate, etc.) for at least 3 months. The time periods were chosen under consideration of the pharmacokinetics (especially elimination half-life) of typically used drugs for treating acute attacks (27) or prophylactic treatment (28).

The only study investigating intentional saccades (anti-gap paradigm) in migraineurs found an increased number of saccade errors in patients with migraine, suggesting a deficit in the inhibition of (unwanted) reflexive saccades (15). Interestingly in the study by Cambron et al. (15), the latency of anti-saccades was in normal range. In contrast, the present study did not find any of the typical consequences of a general deficit in the inhibition of unwanted reflexive saccades: Neither the frequency of erroneous anti-saccades, nor the frequency of suppression errors in the memory-paradigm were increased in the patient group. Furthermore, a suppression deficit would also predict a decrease of the latency of erroneous anti-saccades, which we did not observe (Figure 2B). Saccade latencies in the memory-paradigm were normal. Thus, we could not confirm a general inhibitory deficit as suggested by Cambron et al. (15). The increase of saccade latency that occurred only in the anti-paradigm but not in the memory-paradigm suggests a deficit related to the internal generation of an imagined target (vector inversion).

The neural pathways of vector inversion processes are still widely unclear. Evidence from studies in monkeys have suggested the lateral intraparietal area (LIP) to play a role in vector inversion (29–31). In humans the LIP corresponds to areas of the parietal cortex and indeed recent studies, mainly imaging studies, have suggested areas of the parietal cortex to play a role in anti-saccade generation, possibly vector transformation (32–34). In migraine patients, the frontal lobe and the cerebellum are most commonly described to show structural abnormalities, the parietal lobe also has been shown to be altered in some studies (2, 35). In the pathophysiology of migraine, the parietal cortex is believed to contribute to different migraine symptoms such as visual, motor, and memory functions, as well as cognitive performance (36). Lately there has been some conflicting evidence for a possible impact of migraine on cognition (37). One study found a deficit of sustained attention and processing speed in migraineurs compared to controls in a symbol search task (11). Another study found a missing standard global precedence effect in migraineurs, suggesting a deficit in processing of such global visual features (12). While both these distinct findings cannot explain the specific latency increase in anti-saccades in the present study, they support the possibility of deficits in cortical networks involved in spatial re-mapping in migraine patients. Restrictively it should be noted that the study population of the mentioned studies was much older than in the present study, so that age-effects might be considered in this context.

It could be argued that the results of the present study might point to a distinct, subclinical deficit of functions of parietal cortex areas. Such an effect on parietal function could be mediated in migraineurs by cerebellar parietal projections (38) since cerebellar impairments were repeatedly reported in migraineurs (7). Such functions of the cerebellum in migraine patients should be investigated in more detail in future studies.

## Conclusion

Migraine patients (with and without aura) seem to have a specific deficit in the generation of anti-saccades, indicated by an increased saccade latency. This could suggest a deficit in distinct cortical functions as a vector inversion process mediated through cerebellar cortical projections.

## Data Availability Statement

The raw data supporting the conclusions of this article will be made available by the authors, without undue reservation.

## Ethics Statement

The studies involving human participants were reviewed and approved by Ethics Committee of the Medical Faculty of the Ludwig-Maximilians-University Munich. The patients/participants provided their written informed consent to participate in this study.

## Author Contributions

FF, AS, and TE: conception and study design. FF, CG, FS, and OE: patient recruitment. FF, CG, FS, OE, and TE: data collection. FF and TE: data analysis and writing the article. AS, FS, and OE: critical revision of the article. All authors reviewed and accepted the final manuscript.

## Conflict of Interest

The authors declare that the research was conducted in the absence of any commercial or financial relationships that could be construed as a potential conflict of interest.
